# Gender-Related Differences in the Levels of Ambulatory BP and Intensity of Antihypertensive Treatment in Patients Undergoing Peritoneal Dialysis

**DOI:** 10.3390/life13051140

**Published:** 2023-05-08

**Authors:** Ioannis Kontogiorgos, Panagiotis I. Georgianos, Vasilios Vaios, Georgia Vareta, Eleni Georgianou, Apostolos Karligkiotis, Vasiliki Sgouropoulou, Konstantia Kantartzi, Pantelis E. Zebekakis, Vassilios Liakopoulos

**Affiliations:** 1 2nd Department of Nephrology, AHEPA Hospital, Aristotle University of Thessaloniki, 54636 Thessaloniki, Greece; 2 Department of Nephrology, Democritus University of Thrace, 68100 Alexandroupolis, Greece; 3 Section of Hypertension, 1st Department of Medicine, AHEPA Hospital, Aristotle University of Thessaloniki, 54636 Thessaloniki, Greece

**Keywords:** ambulatory blood pressure, antihypertensive therapy, peritoneal dialysis, sex differences

## Abstract

Prior studies have shown that among patients with chronic kidney disease not yet on dialysis, the faster progression of kidney injury in men than in women is, at least partly, explained by sex differences in ambulatory blood pressure (BP) control. The present study aimed to investigate potential differences in the levels of ambulatory BP and intensity of antihypertensive treatment between men and women with end-stage kidney disease undergoing long-term peritoneal dialysis (PD). In a case-control design, 48 male PD patients were matched for age and heart failure status with 48 female patients in a 1:1 ratio. Ambulatory BP monitoring was performed with an oscillometric device, the Mobil-O-Graph (IEM, Stolberg, Germany). The BP-lowering medications actually taken by the patients were prospectively recorded. No gender-related differences were observed in 24 h systolic BP (129.0 ± 17.9 vs. 128.5 ± 17.6 mmHg, *p* = 0.890). In contrast, 24 h diastolic BP was higher in men than in women (81.5 ± 12.1 vs. 76.8 ± 10.3 mmHg, *p* = 0.042). As compared with women, men were being treated with a higher average number of antihypertensive medications daily (2.4 ± 1.1 vs. 1.9 ± 1.1, *p* = 0.019) and were more commonly receiving calcium-channel-blockers (70.8% vs. 43.8%, *p* = 0.007) and β-blockers (85.4% vs. 66.7%, *p* = 0.031). In conclusion, the present study shows that among PD patients, the levels of ambulatory BP and intensity of antihypertensive treatment are higher in men than in women. Longitudinal studies are needed to explore whether these gender-related differences in the severity of hypertension are associated with worse cardiovascular outcomes for male patients undergoing PD.

## 1. Introduction

Hypertension is an important cause of cardiovascular morbidity and mortality in both the general population and in patients with end-stage kidney disease (ESKD) undergoing long-term peritoneal dialysis (PD) [1,2]. In the general population, the trajectories for blood pressure (BP) with ageing differ between men and women [3]. Hypertension is less frequent in young women than in men of the same age. Thereafter, there is a progressive elevation in the levels of BP in women. At approximately the fourth decade of life, the prevalence of hypertension does not substantially differ between the two genders. However, in the elderly, the severity of hypertension is greater in women than in men [3]. Furthermore, evidence from clinical studies suggests that there are sex differences in the severity of hypertension-mediated target-organ damage. As compared with men, women have been shown to carry a greater risk of developing left ventricular hypertrophy, concentric cardiac remodeling, and subclinical left ventricular diastolic dysfunction [4,5].

Prior studies have shown that there are gender-related differences in the severity of hypertension in patients with chronic kidney disease who are not yet on dialysis [6,7]. For example, in a prospective observational study that enrolled 906 hypertensive patients with predialysis CKD, the worse ambulatory BP control in men than in women at baseline was associated with a higher risk of incident ESKD and all-cause mortality in men over a median follow-up of 10.7 years [8]. It remains unclear whether such gender-related differences in ambulatory BP control exist in patients with ESKD who have even more severe hypertension. Accordingly, the present study aimed to provide a comparison of ambulatory BP levels and intensity of antihypertensive treatment between men and women with ESKD receiving long-term PD.

## 2. Materials and Methods

This was a secondary analysis incorporating data from a cross-sectional study that was conducted in 4 PD centers in Northern Greece aiming to investigate the epidemiology of hypertension in the PD population [2]. Patients were enrolled in the study if they met the following inclusion criteria: (i) ESKD patients who had been treated with continuous ambulatory or automated PD for at least 3 months, and (ii) patients who had signed informed written consents. Patients were excluded from this study in the case of: (i) chronic atrial fibrillation or other arrhythmia; (ii) adjustments to the PD regimen and/or changes in the prescribed antihypertensive medications during the last 2 weeks; (iii) acute peritonitis or other infectious/bleeding complications during the past 1 month; (iv) a body mass index of ≥40 kg/m^2^; (v) the presence of nonfunctioning arteriovenous fistula in both arms that limited the possibility of accurate assessment of BP; and (vi) recent hospitalization for acute coronary syndrome or stroke. All clinical procedures were carried out in accordance with the Declaration of Helsinki and its latest amendments. The study protocol was approved by the Ethics Committee of the School of Medicine, Aristotle University of Thessaloniki (code of approval: 448/18-07-18), and it was registered in http://www.clinicaltrials.gov (accessed on 18 July 2018) (unique identifier: NCT03607747).

For the aims of the present analysis, a member of the investigative team (I.K.), who did not participate in the enrollment process and was unaware of the BP readings, matched at a 1:1 rate 48 male PD patients with 48 female PD patients for age and history of heart failure. All participants visited their PD unit to complete the prespecified protocol procedures. Data on demographic characteristics, medical history, laboratory parameters, antihypertensive treatment, and the prescribed PD regimen were prospectively collected.

Office BP measurements were taken by a nurse trained in this technique, according to the 2018 European Society of Hypertension/European Society of Cardiology (ESC/ESH) guidelines [9]. In all 4 PD centers, office BP was recorded with a validated automated device, the HEM–705 CP (Omron, Healthcare). More specifically, after a 5 min seated rest period, a cuff of appropriate size was fitted to the non-dominant (or non-fistula) arm and 3 BP measurements were performed 1 min apart. These 3 consecutive BP recordings were averaged to provide a standardized office BP measurement.

Ambulatory BP monitoring was performed for 24 h with an oscillometric device, the Mobil-O-Graph (IEM, Stolberg, Germany). Office and ambulatory BP readings were taken from the same arm to avoid inter-arm differences between these 2 techniques. The device was programmed to record ambulatory BP at 20 min intervals in the daytime period (07:00–23:00) and at 30 min intervals in the nighttime period (23:00–07:00). The ambulatory BP monitoring was judged as accurate if >80% of the readings were valid with no more than 2 non-consecutive daytime hours with <2 valid recordings and no more than 1 nighttime hour without a valid BP recording [10].

Hypertension was diagnosed based on at least one of the three following criteria: (i) standardized office BP of ≥140/90 mmHg; (ii) average 24-h ambulatory BP of ≥130/80 mmHg; and (iii) the use of at least 1 antihypertensive agent of any category.

The continuous variables are presented as means ± standard deviations (SDs) or medians and interquartile ranges (IQRs). The categorical variables are reported as frequencies (n) and percentages (%). The Kolmogorov–Smirnov test was used to examine if each variable was normally distributed. Comparisons of the continuous data between males and females were performed with an independent *t*-test or a Mann–Whitney U test, as appropriate. Between-group comparisons of the categorical variables were performed with a Chi-squared test or a Fisher’s exact test. All tests were two-tailed, and a *p*-value of <0.05 was considered statistically significant. The analysis was conducted using the Statistical Package for Social Sciences (SPSS) version 27.0 (SPSS, Chicago, IL, USA).

## 3. Results

**Figure 1:**
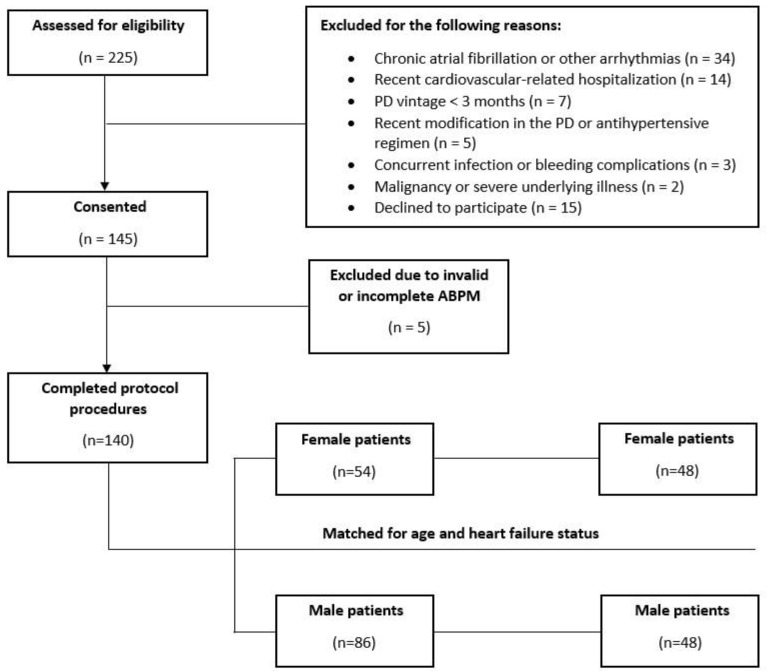
Flow diagram of the patient enrollment process of the study.

The study enrollment procedure is depicted in Figure 1. A total of 225 patients were assessed for eligibility. Of these, 145 patients fulfilled the inclusion/exclusion criteria and provided informed written consent. After the exclusion of 5 patients because of invalid or incomplete ambulatory BP monitoring, a total of 140 patients with complete datasets were finally included in the study. The overall population consisted of 54 female and 86 male PD patients. After the matching procedure for age and heart failure status, 48 pairs of women and men were created. Their basic demographic, clinical, and laboratory characteristics are presented in Table 1. As expected, due to the case-control design of this study, age and history of heart failure did not differ between the female and male participants. As compared with the men, the women had lower body weights (70.5 ± 15.2 vs. 77.9 ± 12.9 kg, *p* = 0.012) and lower levels of serum creatinine (5.9 (4.8–7.6) vs. 8.0 (5.5–13.9), *p* = 0.008). With respect to the mode of PD, the proportion of patients receiving continuous ambulatory PD was higher in the group of women than in the group of men (54.2% vs. 29.2%, *p* = 0.013). There were no statistically significant differences between the two genders in the prevalence of cardiovascular comorbidities, in the smoking status, or in other basic hematological or biochemical parameters (Table 1).

As shown in Table 2, no significant differences between men and women were detected in the office systolic BP and office diastolic BP. The average 24 h ambulatory systolic BPs were similar in both groups (129.0 ± 17.9 vs. 128.5 ± 17.6 mmHg, *p* = 0.890). In contrast, the average 24 h ambulatory diastolic BP was significantly higher in men than in women (81.5 ± 12.1 vs. 76.8 ± 10.3, *p* = 0.042). These gender-related differences were consistent during both the daytime and nighttime periods. In detail, the daytime ambulatory diastolic BP (82.5 ± 11.8 vs. 78.1 ± 10.4, *p* = 0.052) and nighttime ambulatory diastolic BP (78.8 ± 14.0 vs. 73.8 ± 11.1 vs. *p* = 0.056) were higher in men than in women. The office and ambulatory heart rates were similar for the two groups. In addition, volume status, as assessed with the method of bioimpedance spectroscopy, did not significantly differ between the men and the women (Table 2).

There were no significant gender-related differences in the prevalence of hypertension either with the use of office recordings (93.8% vs. 89.6%, *p* = 0.460) or the use of the reference-standard method of ambulatory BP monitoring (93.8% vs. 91.7%, *p* = 0.695) for the diagnosis of hypertension. However, the men were being treated with a higher average number of antihypertensive medications daily (2.4 ± 1.1 vs. 1.9 ± 1.1, *p* = 0.019). Furthermore, as compared with the women, the men were more commonly being treated with calcium channel blockers (70.8% vs. 43.8%, *p* = 0.007) and β-blockers (85.4% vs. 66.7%, *p* = 0.031). In contrast, the male patients were less frequently receiving treatment with mineralocorticoid receptor antagonists than the women (4.2% vs. 20.8%, *p* = 0.014).

## 4. Discussion

The present study showed that among patients undergoing long-term PD, the levels of ambulatory BP were higher in men than in women. In addition, the intensity of antihypertensive treatment, such as the average number of prescribed BP-lowering medications as well as the use of calcium channel blockers and β-blockers, was greater in the male PD patients than in the female PD patients. The findings of the present study are accordant with the results of a prior cross-sectional analysis that compared the rates of ambulatory BP control between 129 male and 91 female patients receiving maintenance hemodialysis [11]. In this study, the 48 h ambulatory systolic BPs (137.2 ± 17.4 vs. 132.2 ± 19.2 mmHg, *p* = 0.045) and 48 h ambulatory diastolic BPs (81.9 ± 12.1 vs. 75.9 ± 11.7 mmHg, *p* < 0.001) were higher in men than in women [11]. Although the prevalence of hypertension did not differ between the two genders, the rates of 48 h ambulatory BP control were significantly lower in the male hemodialysis patients than in the female hemodialysis patients [11]. Similarly, another prior cross-sectional study showed that among kidney transplant recipients, the rates of 24 h ambulatory BP control were significantly lower in the female patients as compared with the male patients (16.9 vs. 30.3%, *p* = 0.029), despite the more intensive use of antihypertensive drug therapy in the men [12]. Taken together, the results of the present study and prior studies show that for the whole spectrum of ESKD, the severity of hypertension is greater in male patients than in female patients.

These sex-related differences in BP levels have biological plausibility. Mechanistic studies have provided evidence that the differences between the two genders in the activation of the renin-angiotensin system (RAS) and the sympathetic nervous system, nitric oxide metabolism as well as the release of sex hormones may mediate the greater severity of hypertension in men than in women [6,13]. Animal studies have shown that there is a higher activation in the AngII–AT1–ACE axis and a faster response to AngII infusion in male hypertensive rats than in female hypertensive rats [14,15]. Additionally, female hormones play a pivotal role in BP regulation. Postmenopausal women have been shown to have higher BP levels whereas the initiation of estrogen replacement therapy is accompanied by improvements in BP profiles [16,17]. Pro-inflammatory T-cells may also explain the sex differences in BP levels, with hypertensive women having more protective immune profiles and lower interleukin-17 levels, resulting in better BP control as compared with men [13,18].

The strength of the present study lies in its careful evaluation of hypertension with the concomitant use of standardized office and ambulatory BP measurements. Unlike the cross-sectional design of prior studies [9,10], an important advantage of the present work was the fact that our study followed a blinded matching procedure in group formation, mitigating the confounding effects of age and history of heart failure on the severity of hypertension. However, there are also some weaknesses that need to be acknowledged. First, the sample size of our study was relatively small; therefore, our analysis may not have been adequately powered to detect statistical significance in the small differences in the office and ambulatory BP levels between the males and females. Second, our study did not follow a longitudinal design and did not explore potential associations between ambulatory BP levels and the risk for adverse cardiovascular events for the two genders. Third, the measurements of the office and ambulatory BP were performed in a single time-point at baseline. Therefore, this study could not evaluate longitudinal changes in the severity of hypertension for the males and females.

Larger and longer-term observational studies are needed to confirm or refute the results of our analysis. If such gender-related differences in the severity of hypertension truly exist, then the worse ambulatory BP measurements in men might be a plausible mechanistic explanation for the sex differences in cardiovascular outcomes among patients receiving kidney replacement therapy.

## Figures and Tables

**Table 1:** Demographic, clinical, and laboratory characteristics of the male and female PD patients.

Parameter	Men (n = 48)	Women (n = 48)	*p*-Value
Age (years)	62.5 (54.0–70.5)	62 (53.3–70.8)	0.956
Weight (kg)	77.9 ± 12.9	70.5 ± 15.2	0.012
BMI (kg/m^2^)	26.6 ± 4.0	27.5 ± 5.9	0.356
Time on PD (months)	21.0 (6.0–39.0)	21.5 (7.0–44.8)	0.867
Continuous ambulatory PD (n, %)	14 (29.2%)	26 (54.2%)	0.013
Peritoneal ultrafiltration (L)	0.6 (0.4–1.0)	0.8 (0.5–1.0)	0.412
Dialysate-to-plasma creatinine ratio	0.69 ± 0.10	0.67 ± 0.11	0.232
Peritoneal transport status (n, %)			
Low	3 (6.3%)	6 (12.5%)	
Low-average	13 (27.1%)	13 (27.1%)	
High-average	27 (56.3%)	23 (47.9%)	
High	5 (10.4%)	6 (12.5%)	
Residual diuresis ≥ 0.5 L/day (n, %)	35 (72.9%)	33 (68.8%)	0.653
Comorbidities (n, %)			
Diabetes mellitus	20 (41.7%)	18 (37.5%)	0.676
Dyslipidemia	33 (68.8%)	33 (68.8%)	1.000
Coronary artery disease	16 (33.3%)	11 (22.9%)	0.256
Peripheral vascular disease	8 (16.7%)	3 (6.3%)	0.109
Heart failure	9 (18.8%)	9 (18.8%)	1.000
Current smokers (n, %)	11 (22.9%)	7 (14.6%)	0.296
Laboratory parameters			
Hemoglobin (g/dL)	11.5 (10.9–12.6)	11.2 (10.3–11.9)	0.065
Serum urea (mg/dL)	123.9 ± 34.8	112.7 ± 32.4	0.106
Serum creatinine (mg/dL)	8.0 (5.5–13.9)	5.9 (4.8–7.6)	0.008
Serum albumin (g/dL)	3.9 (3.5–4.0)	3.7 (3.6–4.0)	0.371
Serum sodium (mEq/L)	138 (137–140)	138 (136–140)	0.474
Serum potassium (mEq/L)	4.5 ± 0.6	4.4 ± 0.6	0.516

Abbreviations: BMI = body mass index; PD = peritoneal dialysis. The continuous data are presented as means ± SDs or medians (IQRs).

**Table 2:** Blood pressure measurements, volume statuses, and antihypertensive agents used in treating the male and female PD patients.

Parameter	Men (n = 48)	Women (n = 48)	*p*-Value
Office BP			
Systolic (mmHg)	135.5 ± 21.0	134.4 ± 18.2	0.768
Diastolic (mmHg)	79.6 ± 11.6	80.1 ± 14.1	0.856
Ambulatory 24 h BP			
Systolic (mmHg)	129.0 ± 17.9	128.5 ± 17.6	0.890
Diastolic (mmHg)	81.5 ± 12.1	76.8 ± 10.3	0.042
Ambulatory daytime BP			
Systolic (mmHg)	129.6 ± 17.6	129.7 ± 17.5	0.991
Diastolic (mmHg)	82.5 ± 11.8	78,1 ± 10.4	0.052
Ambulatory nighttime BP			
Systolic (mmHg)	127.0 ± 19.8	125.9 ± 18.8	0.776
Diastolic (mmHg)	78.8 ± 14.0	73.8 ± 11.1	0.056
HR (bpm)			
Office HR	73.7 ± 9.8	74.9 ± 11.7	0.573
Ambulatory 24 h HR	72.4 ± 8.1	72.3 ± 9.9	0.964
Ambulatory daytime HR	73.5 ± 0.3	73.6 ± 10.7	0.949
Ambulatory nighttime HR	69.6 ± 8.6	69.0 ± 9.1	0.730
Prevalence of hypertension			
Ambulatory BP ≥ 130/80 mmHg or antihypertensive drug use (n, %)	45 (93.8%)	44 (91.7%)	0.695
Office BP ≥ 140/90 mmHg or antihypertensive drug use (n, %)	45 (93.8%)	43 (89.6%)	0.460
Volume status			
BIS-derived overhydration index (L)	1.7 (0.1–3.3)	0.8 (−0.3–1.9)	0.071
Overhydration index > 2.5 L (n, %)	16 (33.3%)	8 (16.7%)	0.059
Patients treated with antihypertensives (n, %)	45 (93.8%)	42 (87.5%)	0.294
Number of antihypertensive medications (n, %)	2.4 ± 1.1	1.9 ± 1.1	0.019
Antihypertensive agent classes			
ACEIs/ARBs	25 (52.1%)	22 (45.8%)	0.540
CCBs	34 (70.8%)	21 (43.8%)	0.007
β-blockers	41 (85.4%)	32 (66.7%)	0.031
α-blockers	6 (12.5%)	1 (2.1%)	0.111
MRAs	2 (4.2%)	10 (20.8%)	0.014
Central acting agents	7 (14.6%)	3 (6.3%)	0.181

Abbreviations: BIS = bioimpendence spectroscopy; bpm = beats per minute; ACEI = angiotensin-converting enzyme inhibitor; ARB = angiotensin receptor blocker; BP = blood pressure; CCB = calcium channel blocker; HR = heart rate; MRA = mineralocorticoid receptor antagonist.

## Data Availability

The data used in this study are not publicly available due to privacy and ethical concerns. These data may be shareable after contacting the corresponding author.
